# Genomic Characterization of Multidrug-Resistant *Acinetobacter baumannii* in Pneumonia Patients in Kazakhstan

**DOI:** 10.3390/diagnostics15060704

**Published:** 2025-03-12

**Authors:** Vitaliy Strochkov, Vyacheslav Beloussov, Shynggys Orkara, Alyona Lavrinenko, Maxim Solomadin, Sergey Yegorov, Nurlan Sandybayev

**Affiliations:** 1 Kazakhstan-Japan Innovation Centre, Kazakh National Agrarian Research University, Almaty 050010, Kazakhstan; 2 Molecular Genetic Laboratory TreeGene, Almaty 050008, Kazakhstan; 3 Scientific Research Laboratory, Karaganda Medical University, Karaganda 100000, Kazakhstan; 4 Department of Biology, School of Sciences and Humanities, Nazarbayev University, Astana 010000, Kazakhstan

**Keywords:** *A. baumannii*, whole-genome sequencing, AMR, multilocus sequence typing

## Abstract

**Background/Objectives**: *Acinetobacter baumannii* is an increasingly significant nosocomial pathogen causing severe infections globally. The emergence of multidrug-resistant *A. baumannii* strains has raised concerns about the efficacy of current treatment options. This study aimed to investigate the molecular epidemiology and antimicrobial resistance patterns of *A. baumannii* isolates from Kazakhstan. **Methods**: We collected nine *A. baumannii* isolates in 2022–2023 in Karaganda, Kazakhstan, which were then subjected to whole-genome sequencing (WGS) using the IonTorrent platform for genome characterization. Multilocus sequence typing (MLST) was used to classify the isolates into distinct clonal complexes. In addition, antibiotic susceptibility testing was conducted using the standard methods for a range of antibiotics commonly used against *A. baumannii*. **Results**: Our results revealed a high degree of genomic diversity among isolates from Kazakhstan, with multiple distinct classes identified: ST78 (*n* = 4, 44.4%), ST15 (*n* = 2, 22.2%), ST2 (*n* = 2, 22.2%), and ST193 (*n* = 1, 11%). MLST analysis showed that ST78^Pas^/1104^Oxf^ (harboring blaOXA-72 and blaOXA-90 genes) were prevalent among the multidrug-resistant isolates. Based on the results of MLST, KL, and OCL, the analyzed isolates were assigned to specific international clones: IC2—ST2(Pas)-KL2/168-OCL1, IC4—ST15(Pas)-KL9-OCL7, and IC6—ST78(Pas)-KL49-OCL1. Notably, these isolates exhibited resistance to multiple antibiotics including meropenem, imipenem, gentamicin, amikacin, and ciprofloxacin. **Conclusions**: This study highlighted the complex molecular epidemiology of *A. baumannii* in Kazakhstan over a two-year period, underscoring the need for targeted surveillance strategies to monitor antimicrobial resistance patterns. The emergence and dissemination of multidrug-resistant strains within this timeframe emphasizes the importance of whole-genome sequencing as a diagnostic tool and underscores the challenges posed by these infections.

## 1. Introduction

*Acinetobacter baumannii* is an aerobic, Gram-negative coccobacillus responsible for healthcare-associated infections [1]. *A. baumannii* has emerged as a leading cause of hospital-acquired or nosocomial infections, including pneumonia, meningitis, bloodstream infections, and urinary tract infections, leading to considerable morbidity and mortality among immunocompromised patients [2]. *A. baumannii*-related infections account for approximately 20% of the infections in intensive care units, representing the majority of clinically relevant strains [3,4,5].

Considering the severity of the infections, their level of resistance, and the limited treatment options, the World Health Organization (WHO) listed carbapenem-resistant *A. baumannii* as the top “critical” priority pathogen to enhance global preparedness and accelerate antibiotic discovery [6].

*A. baumannii* exhibits a high degree of resistance to existing antimicrobial classes, particularly carbapenems and colistin, which are considered last-resort treatments [7].

Among the diverse resistance mechanisms, the production of carbapenemases, particularly class D β-lactamases (oxacillinases), is one of the most common mechanisms for carbapenem resistance in carbapenem-resistant *A. baumannii*. Oxacillinases, such as blaOXA-23, blaOXA-24, and blaOXA-58, are frequently found among *A. baumannii* strains. These enzymes are encoded by various alleles with differing hydrolytic capacities [8]. In Kazakhstan, 82.2% of *A. baumannii* strains were found to harbor acquired carbapenemase genes, including blaOXA-23 (78.6%) and blaOXA-58 (3.6%) [9].

The widespread dissemination of multidrug-resistant (MDR) *A. baumannii* (MDRAB) strains poses a substantial public health threat globally. A meta-analysis determined the worldwide prevalence of MDRAB in hospital-acquired pneumonia or ventilator-associated pneumonia patients to be approximately 79.9%, with mortality rates ranging from 37.2% to 48.1% [10]. This phenomenon is further complicated by the complex interplay of various virulence factors that contribute to the pathogenicity of MDRAB strains, significantly contributing to high mortality rates and posing a severe challenge for clinicians [11]. Evidence suggests that specific and nonspecific virulence factors play critical roles in key processes such as adhesion, cytotoxicity, immune evasion, microbial interaction, genetic rearrangements, and biofilm formation [12,13]. The ability of MDRAB to form robust biofilms is particularly concerning, because it enables these bacteria to persist in healthcare environments, evade host immune responses, and develop increased resistance to antimicrobial agents. Biofilm formation also facilitates their adherence to various surfaces and survival in harsh conditions, while microbial interactions within these communities further enhance their antimicrobial resistance mechanisms [14]. The complex interplay between biofilm architecture and antibiotic resistance mechanisms underscores the urgent need for comprehensive research into the underlying processes driving *A. baumannii* infections [15].

Understanding the mechanisms of acquired antimicrobial resistance (AMR), virulence-associated genes, and the genomic diversity among MDRAB isolates is essential for elucidating their dissemination patterns within regions. Whole-genome sequencing (WGS) allows for a detailed examination of the genomic composition of *A. baumannii*, beyond traditional phenotypic and genotypic characterization [16].

This study aimed to characterize the genomic features of MDR *A. baumannii* isolates recovered from pneumonia patients in Kazakhstan. By analyzing the whole-genome sequences of these isolates, we sought to identify the molecular mechanisms underlying their drug resistance and virulence, as well as the correlation between capsular locus (KL), sequence type (ST), and antibiotic resistance genes. Our findings offer valuable insights into the epidemiology and evolution of *A. baumannii* in Kazakhstan.

## 2. Materials and Methods

### 2.1. Isolation and Identification of Acinetobacter baumannii

*A. baumannii* isolates were obtained from the sputum samples of patients with pneumonia from three clinical facilities in Karaganda, Kazakhstan from 2022 to 2023. The isolates were grown on MacConkey agar (Oxoid Limited, Basingstoke, UK), and identified by matrix-assisted laser desorption/ionization–time-of-flight mass spectrometry (MALDI-TOF MS) using the Microflex LT system and the MALDI Biotyper Compass v.4.1.80 software (Bruker Daltonics, Hamburg, Germany).

### 2.2. Antimicrobial Susceptibility Testing

The determination of sensitivity to antimicrobial drugs (amikacin, gentamicin, imipenem, meropenem, ciprofloxacin, etc.) was carried out by the disk diffusion test in Mueller–Hinton agar and by the microdilution method (colistin) in Mueller–Hinton broth. The interpretation of susceptibility testing results was performed according to the European Committee on Antimicrobial Susceptibility Testing guidelines (EUCAST v 13.1) [17]. The quality control of the sensitivity determination was performed using *Escherichia coli* ATCC^®^ 25922, *Escherichia coli* ATCC^®^ 35218, and *Pseudomonas aeruginosa* ATCC^®^ 27853.

The analysis of the results for determining sensitivity to antimicrobial drugs was carried out using WHONET 2022 software and the online platform AMRcloud [18].

### 2.3. Whole Genome Sequencing and Analysis

The genomic DNA of 9 *Acinetobacter* isolates were extracted using PureLink^TM^ Genomic DNA Mini Kit (Invitrogen, Waltham, MA, USA) according to manufacturer’s instructions. The DNA library preparation was constructed by using Ion Xpress^TM^ Plus Fragment Library Kit (ThermoFisher, Waltham, MA, USA). Whole genome sequencing was conducted on an Ion GeneStudio S5 Sequencer platform (ThermoFisher, Waltham, MA, USA).

The quality and trimming of the raw reads were assessed using FaQCs “https://github.com/LANL-Bioinformatics/FaQCs (accessed on 20 December 2024)” [19].

The quality filtered reads were assembled using SPAdes v.3.15.5 [20]. The assembly characteristics and completeness of the draft genome were estimated using QUAST v.5.2.0 [21]. PROKKA v.1.14.6 was used to annotate all assembled sequences [22].

Multi-locus sequence typing (MLST) was performed using mlst software “https://github.com/tseemann/mlst (accessed on 20 December 2024)” with both the University of Oxford [23] and the Institute Pasteur [24] schemes.

Core genome multi-locus sequence typing (cgMLST) was performed using cgMLSTFinder v.1.2 “https://cge.food.dtu.dk/services/cgMLSTFinder/ (accessed on 20 December 2024)”.

In addition, the capsular polysaccharide (K locus) and lipooligosaccharide (OC locus) of *A. baumannii* were predicted using command-line Kaptive 3.0 [25].

### 2.4. In Silico Screening of Antibiotic Resistance and Virulence Genes

The AMR genes were identified using ABRicate “https://github.com/tseemann/abricate (accessed on 20 December 2024)” against the Comprehensive Antimicrobial Resistance Database (CARD) [26]. The draft genomes were scanned against the VFDB database [27] to identify the virulence genes.

### 2.5. Phylogenetic Analyses

We used Roary [28] to perform the pan-genome analysis of 13 *A. baumannii* genomes, including our 9 isolates, using the gff3 files generated by Prokka at a 95% minimum blastp identity and 99% core definition threshold. Then, a maximum-likelihood phylogenetic tree was built using FastTree 2.1.11 [29]. The resulting phylogenetic tree was visualized using TreeViewer v.2.2.0 [30].

## 3. Results

### 3.1. Identification of Bacterial Isolates and Antibiotic Susceptibility Testing

A total of 50 clinical samples were collected from patients with pneumonia across the three healthcare facilities in Karaganda (hem—hematological center, kar—cardiology center, and unc—medical university clinic). Among these, nine isolates (18%) were identified as *Acinetobacter baumannii*. The characteristics of these isolates are presented in Table 1.

**Figure 1:**
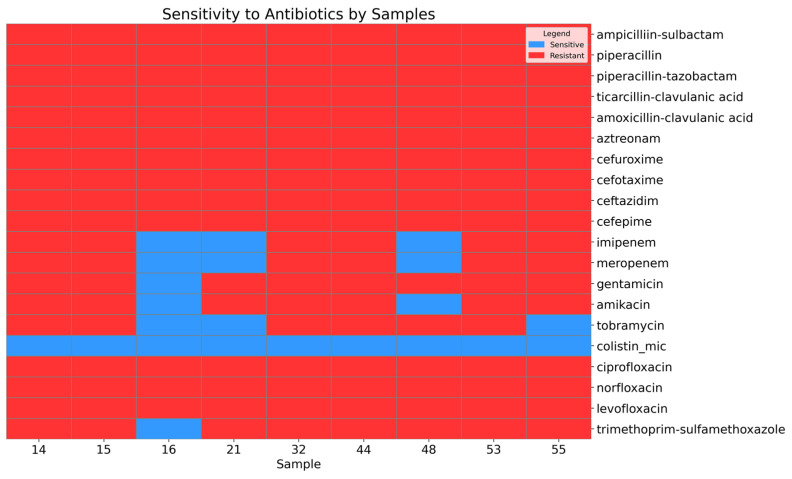
Heatmap showing the sensitivity and resistance of the *A. baumannii* isolates to antibiotics.

A high level of antimicrobial resistance was observed in the *Acinetobacter baumannii* isolates (Figure 1).

All isolates were resistant to the penicillin beta-lactam and cephalosporin classes. Among the isolates, 66.67% (95% CI: 35.42–87.94) were resistant to carbapenem antibiotics, including imipenem and meropenem. Similarly, 66.67% (95% CI: 35.42–87.94) of the isolates were resistant to tobramycin, while 77.78% (95% CI: 45.26–93.68) exhibited resistance to amikacin, and 88.89% (95% CI: 56.5–98.01) were resistant to gentamicin. Antimicrobial agents from the fluoroquinolone class, including levofloxacin and ciprofloxacin, demonstrated a high level of resistance (95% CI: 70.09–100). Additionally, 88.89% (95% CI: 56.5–98.01) of the isolates were resistant to trimethoprim–sulfamethoxazole. However, sensitivity to the last-resort antibiotic colistin was preserved (95% CI: 70.09–100).

### 3.2. Whole-Genome Sequencing and Pan-Genome Analysis

The whole-genome sequencing of the nine *A. baumannii* isolates collected in Kazakhstan during 2022–2023 yielded an average of 2,054,488 reads per isolate (range: 1,238,427–3,801,619). The average sequencing coverage was 55.42× (range: 30.51×–106.31×).

For comparative analysis, four isolates collected in Kazakhstan in 2016 were included: KZ-1106 (GenBank ID—JAUMSL000000000), KZ-1093 (JAJAWC000000000), KZ-1101 (JAPYKX000000000), and KZ-1257 (JALDNC000000000). The reference genome used for comparison was the strain ATCC-19606 (CP045110).

The genome assemblies had an average genome size of 3,941,951 bp (range: 3,823,224–4,065,686 bp) with an average GC content of 38.98%. The average N50 value for the 13 Kazakhstan isolates was 78,743 bp (range: 37,164–132,398 bp) (Table S1).

MLST analysis was performed using two schemes: Pasteur (Pas) and Oxford (Oxf). The isolates collected in 2022–2023 were divided into four distinct classes. Four isolates were identified as ST78^Pas^/ST1104^Oxf^, two isolates as ST15^Pas^/ST236^Oxf^, and two isolates were classified as ST2^Pas^; however, they were divided into two different groups according to the Oxford scheme (ST450 and ST452). The one isolate was identified as ST193^Pas^/ST1110^Oxf^.

The isolates collected in 2016 also belonged to three different groups based on the Pasteur scheme: ST106, ST1574, and ST498 (Table 2). Some genes could not be accurately typed using available sequences, preventing the assignment of a definitive sequence type to these isolates.

Kaptive-based identification and typing of *A. baumannii* predicted that the thirteen draft genomes belonged to nine capsular (K locus) and five polysaccharide (O locus) types. Based on the results of MLST, KL, and OCL, the analyzed isolates were assigned to specific international clones: IC2—ST2^Pas^-KL2/168-OCL1 (*n* = 2, 15.3%), IC4—ST15^Pas^-KL9-OCL7 (*n* = 2, 15.3%), and IC6—ST78^Pas^-KL49-OCL1 (*n* = 4, 30.7%).

The pan-genome consisted of 8470 genes, which were distributed into 2214 core (99% ≤ strains ≤ 100%), 2443 shell (15% ≤ strains ≤ 95%), and 3813 cloud genes (0% ≤ strains ≤ 15%).

**Figure 2:**
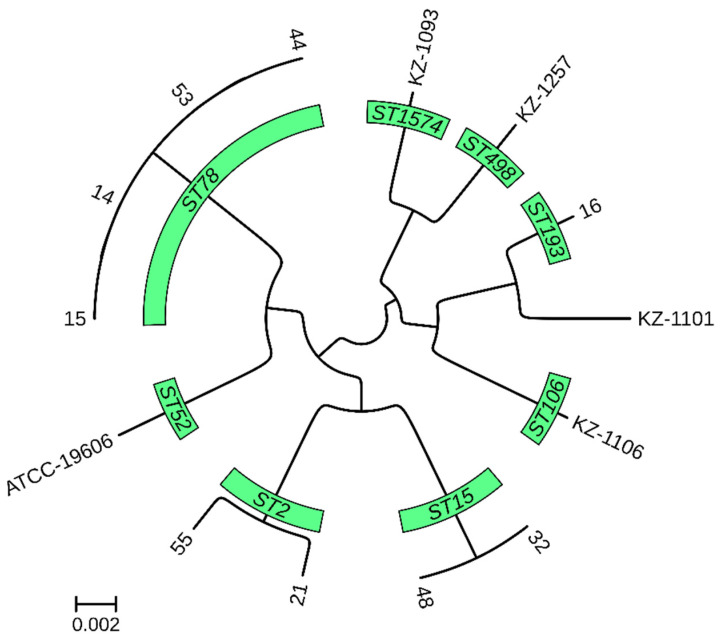
Core genome phylogenetic tree of 13 *A. baumannii* genomes from Kazakhstan.

A pan-genome-based phylogenetic tree revealed the distinct clustering of isolates according to their MLST, cgMLST, and K locus typing profiles (Figure 2).

The tree represents the evolutionary relationship among the isolates, derived from whole-genome comparisons. The branch lengths indicate the degree of genetic divergence between strains. The isolates are grouped according to their MLST profiles.

### 3.3. Antimicrobial Resistance Determinants

The comparison of antibiotic resistance gene profiles revealed that isolates collected in 2022–2023 possessed more genes conferring antibiotic resistance compared to those isolated in 2016.

**Figure 3:**
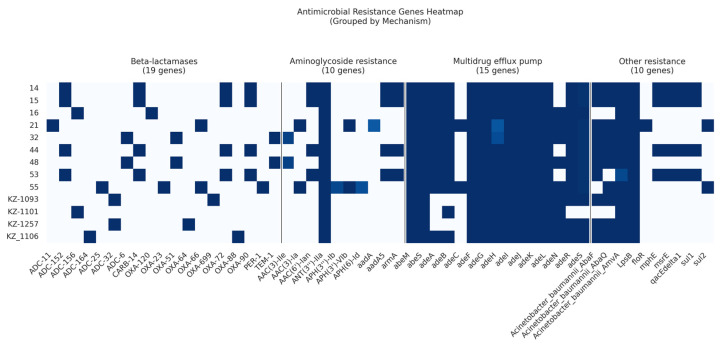
Heatmap showing the presence or absence of acquired AMR genes within the *A. baumannii* draft genomes.

Among the 13 analyzed isolates, we identified a total of 19 β-lactamase resistance genes, 10 AMR genes conferring resistance to aminoglycosides, and 2 macrolide resistance genes (Figure 3).

Three Ambler classes of β-lactamases (classes A, C, and D) were identified in the current study.

Carbapenem-hydrolyzing class D β-lactamases were prevalent in 12 out of 13 isolates (92%). Specifically, four isolates belonging to ST78^Pas^ harbored two genes: blaOXA-72 and blaOXA-90. Two isolates classified as ST15^Pas^ contained the gene blaOXA-51. In contrast, other isolates exhibited a range of Class D β-lactamases represented by blaOXA-120, blaOXA-23, blaOXA-64, blaOXA-66, blaOXA-699, and blaOXA-88 (Table S2).

Class A β-lactamases were also identified: blaCARB-14 was present in four (30.7%) isolates, blaTEM-1 in two isolates, and blaPER-1 in a single isolate.

Seven cephalosporinase ADC variants of Class C β-lactamases were detected. Specifically, four isolates harbored the ADC-152 variant, while two isolates contained either the ADC-156 or ADC-32/ADC-6 variants. The remaining ADC variants identified included ADC-11, ADC-164, and ADC-25.

Aminoglycoside-modifying enzymes, including acetyltransferases (AACs), methyltransferase (armA), phosphotransferases (APHs), and nucleotidyltransferases (ANTs), were identified. The new subclass of intrinsic aminoglycoside nucleotidyltransferase, ANT(3′′)-IIa, was widely distributed and was found in all strains, while the intrinsic aminoglycosides O-phosphotransferase aph(6)-Id and aph(3′′)-Ib were found in only one isolate. In the current study, AAC genes were detected in 61% of the isolates. Moreover, the intrinsic aminoglycoside methyltransferase armA was found in four (30.7%) isolates.

The gene encoding resistance to florfenicol, floR, was identified in a single isolate.

Two macrolide resistance genes, mphE and msrE, were found in four isolates belonging to ST78^Pas^.

Two genes encoding resistance to sulfonamides, sul1 and sul2, were identified in four (30.7%) and two (15%) isolates, respectively.

Our study highlights the complex mechanisms underlying antibiotic resistance in *A. baumannii* isolates from Kazakhstan. Specifically, we identified four categories of efflux pumps: the RND (resistance-nodulation-division) superfamily, the MFS (major facilitator superfamily), the MATE (multidrug and toxic compound extrusion) family, and the SMR (small multidrug resistance) family transporters.

Interestingly, the most prevalent efflux pumps were found to be members of the MFS transporter family (amvA), RND superfamily (adeFGH, adeIJK, and adeL), SMR family (abeS), and MATE family (abeM). These pump-coding genes were consistently detected in all isolates examined. RND efflux pump-coding genes (adeN, adeR, adeS, and adeAB) were found in 69.2% to 92.3% of the isolates (Table S2).

The ubiquity of these efflux pumps underscores the significance of this mechanism in conferring antibiotic resistance in A. baumannii populations. Our findings have implications for understanding the molecular epidemiology of antimicrobial resistance and highlight potential targets for therapeutic intervention.

### 3.4. Virulence-Associated Genes

The investigation of the virulence factors of *Acinetobacter* spp. in the MDRAB isolates revealed the presence of virulence genes associated with adherence, biofilm formation, enzyme, immune evasion, iron uptake, regulation, and serum resistance.

All the genomes carried the barA, barB (sideophore efflux system), basABCDFGHIJ, bauBCDEF, entE (acinetobactin biosynthesis and intake), plcC (phospholipase), OmpA (outer membrane protein A), AdeFGH efflux pump, PANG (poly-N-acetylglucosamine), LPS (lipopolysaccharide), T2SS (Type II secretion system), and PbpG (penicillin-binding protein) genes.

The biofilm-associated protein (bap) gene was detected in eight isolates (61.5%). Eleven isolates (84.6%) carried all the genes of the Csu fimbriae, while two isolates lacked the csuA, csuA/B, and csuB genes. This study revealed that four (30.7%) and three (23.1%) isolates harbored virulence factors, including the hemO cluster and quorum sensing systems. The virulence-associated gene results are shown in Table S3.

## 4. Discussion

The rapid increase in MDRAB infections poses a serious threat to global health. Since most MDRAB isolates exhibit resistance to multiple antimicrobial classes, effectively managing these infections has become increasingly challenging, if not impossible, using available antibiotics. In response to these challenges, researchers worldwide are working to characterize and understand the mechanisms underlying antimicrobial resistance in MDRAB isolates. To contribute to these efforts, we employed whole-genome sequencing (WGS) as a comprehensive approach to investigate the genetic basis of resistance in nine MDRAB isolates collected from hospitals in Kazakhstan.

Our study revealed that the largest proportion of MDRAB isolates belonged to ST78, which is classified as part of International Clone 6 (IC6). Two isolates were assigned to ST15 (IC4) and two to ST2 (IC2), while one isolate did not cluster within any known international clone [31].

The IC6 clonal lineage, to which the ST78 (Pasteur scheme) representatives belong, is a recently emerged lineage. The *A. baumannii* ST78 strain was first identified in Italy in 2006 [32], with subsequent reports of this genotype in the USA, Brazil [33], France [34], and Russia [35]. Whole-genome sequencing of the isolates identified the following antibiotic resistance genes: ADC-152 (cephalosporin), blaCARB-14, blaOXA-72, and blaOXA-90 (carbapenem); AAC(6′)-Ian, aadA5, and armA (aminoglycosides); mphE and msrE (macrolides); as well as qacEDelta1 and sul1, which contribute to the resistance to disinfectants and sulfonamides, respectively.

Multidrug-resistant (MDR) carbapenemase-producing ST15^Pas^ (IC4) *A. baumannii* strains are endemic in South America [36], but they are also present in Europe [37]. The antimicrobial resistance (AMR) genes identified in these isolates included ADC-6, blaOXA-51, blaTEM-1, and AAC(3)-IIe. The presence of blaTEM-1, a class A β-lactamase gene, is notable, as it is frequently associated with extended-spectrum β-lactamase (ESBL) activity, contributing to resistance against cephalosporins and penicillin.

ST2 (IC2) strains are recognized as the dominant carbapenem-resistant *A. baumannii* strains worldwide [38]. However, our study revealed a decrease in the prevalence of IC2 strains compared to previous years [9]. Despite their declining prevalence, the ST2 isolates in our study exhibited a broad range of resistance determinants, underscoring their capacity to persist in clinical settings. Both isolates within this group harbored common resistance genes, including blaOXA-66, aac(3)-Ia, aph(3′)-VIb, adeC, and sul-2, which contribute to resistance against carbapenems, aminoglycosides, and sulfonamides. However, some resistance genes varied between the isolates: isolate #21 contained ADC-11, aadA, and floR, while isolate #55 possessed ADC-25 and additional carbapenem resistance genes (blaOXA-23 and blaPER-1), suggesting a higher level of carbapenem resistance in this isolate. These findings reinforce the heterogeneity of resistance profiles among ST2 isolates and emphasize the need for continued genomic surveillance to track their evolution and adaptation.

All isolates examined were characterized by the presence of resistance genes ANT(3′′)-IIa, abeM, and abeS, as well as genes encoding the efflux pumps adeFGH and adeIJK. Efflux pumps play a critical role in the development of antimicrobial resistance in *A. baumannii.* The adeFGH and adeIJK pumps have been previously associated with resistance to fluoroquinolones, aminoglycosides, β-lactams, and tetracyclines [39].

The high degree of genomic diversity observed among the isolates indicates that *A. baumannii* populations are rapidly evolving and adapting to changing selective pressures, particularly antibiotic use [40].

Beyond their resistance to antimicrobials, pathogenic bacteria have also undergone evolution to counter host defense mechanisms, thereby enhancing their virulence capabilities [41,42]. Notably, our analysis revealed that all *A. baumannii* isolates harbored a range of virulence-associated genes. These genes are responsible for encoding various virulence factors, including those involved in adhesion, biofilm formation, enzymatic activity, immune system evasion, iron acquisition, regulatory processes, and resistance to serum-mediated killing. Furthermore, the genomes exhibited varying degrees of the presence of genes encoding the biofilm-associated protein (Bap) and Csu pili, which are known to contribute to motility, adhesion, and biofilm formation. Notably, Bap plays a crucial role in mediating biofilm formation and maturation, and its involvement in intercellular adhesion has been linked to nosocomially acquired device-related *A. baumannii* infections [43]. Additionally, the Csu pilus is a key virulence factor that facilitates biofilm formation and promotes disease progression by enhancing bacterial adhesion to the epithelial cells [44]. Another significant VF of *A. baumannii* is the outer membrane protein A (OmpA), which has been associated with antibiotic resistance, adherence, and the invasion of host cells, as well as the stimulation of the immune response [45]. Our findings indicate that the ompA gene is widely distributed among *A. baumannii* strains in Kazakhstan, as it was present in all analyzed genomes. The presence of these virulent genes in isolates may contribute to an increased potential for pathogenicity and a heightened severity of infection [42]. Furthermore, the possibility exists that these virulence genes could be disseminated to other bacterial species through horizontal gene transfer, a mechanism similar to that observed with antimicrobial resistance genes [41].

Our findings have significant implications for public health policy in Kazakhstan, emphasizing the urgent need for targeted interventions to control the spread of MDR *A. baumannii* infections. Whole-genome sequencing (WGS) is an invaluable tool for outbreak investigations and antibiotic stewardship efforts [46]. The integration of whole-genome sequencing (WGS) into diagnostic and public health laboratories has the potential to revolutionize our understanding of pathogens by enabling the more precise identification, typing, antimicrobial susceptibility testing, and determination of pathogenicity [47]. Currently, when initiating or selecting antimicrobial therapy based on phenotypic antimicrobial susceptibility testing (AST), clinicians lack routine information about the potential threat posed by isolated pathogens. However, future WGS data linking specific genetic determinants to adverse clinical outcomes may significantly influence chemotherapy strategies [48]. For instance, identifying highly pathogenic strains could help to identify patients at higher risk of infection-related complications, prompting more aggressive or combination therapy and prolonged intravenous antimicrobial treatment. Conversely, if WGS reveals that certain potential pathogens are relatively harmless, this knowledge could facilitate the use of less intensive treatment approaches, such as shorter durations of therapy, oral medications, reduced patient monitoring, and fewer diagnostic investigations, ultimately leading to earlier hospital discharge.

In a broader context, our study contributes to the growing body of evidence on the global dissemination of MDR *A. baumannii* clones. The results underscore the importance of coordinated international efforts to monitor and control antimicrobial use, reducing the spread of resistant strains.

Finally, we conclude that collecting comprehensive data on the dissemination of specific strains, high-risk clones, antimicrobial resistance, and virulence factors across hospitals, countries, and regions is crucial. This information will aid in developing epidemiological strategies to prevent the exponential spread of MDR *A. baumannii* and other pathogenic bacterial species.

## Figures and Tables

**Table 1:** Characterization of the *A. baumannii* isolates.

Sample	Gender	Age	Facility	Date of Isolation
14	female	66	hem	12/2022
15	male	60	unc	12/2022
16	male	58	unc	12/2022
21	male	61	unc	3/2023
32	male	60	unc	6/2023
44	male	72	unc	9/2023
48	female	53	unc	10/2023
53	male	79	unc	10/2023
55	male	71	kar	10/2023

**Table 2:** Comprehensive typing profiles for *A. baumannii* isolates.

cgMLST	K Locus	O Locus	ST(Pas)	ST(Oxf)	Location	Sample
777	KL49	OCL1	78	1104	Karaganda	14
777	KL49	OCL1	78	1104	Karaganda	15
998	KL52	OCL6	193	1110	Karaganda	16
459	KL168	OCL1	2	450	Karaganda	21
856	KL9	OCL7	15	236	Karaganda	32
777	KL49	OCL1	78	1104	Karaganda	44
856	KL9	OCL7	15	236	Karaganda	48
777	KL49	OCL1	78	1104	Karaganda	53
456	KL2	OCL1	2	452	Karaganda	55
976	KL141	OCL5	106	2216	Karaganda	KZ-1106
873	KL128	OCL12	1574	-	Astana	KZ-1093
994	KL37	OCL6	-	-	Astana	KZ-1101
1000	KL127	OCL6	498	-	Astana	KZ-1257

## Data Availability

The raw data have been submitted to NCBI. The project accession number is PRJNA1165446 (SRR31226071-79).

## Supplementary Materials

The following supporting information can be downloaded at: https://www.mdpi.com/article/10.3390/diagnostics15060704/s1, Table S1: The data of the genome assembly of 13 *A. baumannii* isolates from Kazakhstan; Table S2: Predicted AMR genes; and Table S3: Putative virulence factor genes.
